# Analysis of Sexual Inhibition and Satisfaction from a Gender Perspective among University Students

**DOI:** 10.3390/ijerph18157994

**Published:** 2021-07-28

**Authors:** Samuel P. León, Cristina Abengózar Sánchez, José María Augusto-Landa, Inmaculada García-Martínez

**Affiliations:** 1 Department of Pedagogy, University of Jaén, 23071 Jaén, Spain; sparra@ujaen.es (S.P.L.); cas00015@red.ujaen.es (C.A.S.); 2 Department of Psychology, University of Jaén, 23071 Jaén, Spain; 3 Department of Didactics and School Organization, University of Granada, 18071 Granada, Spain

**Keywords:** sexual violence, university students, sexual inhibition, sexual satisfaction, sex role

## Abstract

University is characterized by a critical stage where students experience their sexuality, across a range of relationships. From these experiences, university students consolidate their personality and their sexual role. Factors such as age, sex, or traumatic experiences of violence or sexual abuse can affect their sexual role. The present study aims to analyze how the variables age, sex and having suffered abuse or violence may predict sexual satisfaction and inhibition. In addition, we analyze the mediating effect that sexual role plays on these relationships. For this purpose, Bem Sex Role Inventory (BSRI-12), Sociosexual Orientation Inventory (SOI-R), Inhibited Sexual Desire Test (ISD) and New Sexual Satisfaction Scale (NESS) were administered to 403 university students. The findings report that sex (β = −0.313), age (β = −0.116) and being a survivor of sexual assault (β = 0.413) are predictive of male role, but not from the female role. Also, people with more male features tend to have lower levels of commitment and inhibition than those who have more female ones.

## 1. Introduction

Currently, sex and gender terms are used indiscriminately, although their meanings are different. The knowledge of these concepts helps to understand that the differences between men and women are not just purely biological [1]. In this regard, sex addresses the biological and anatomical differences that exist between women and men, which refers merely to physical features of the body, as well as sexual activity. Gender, on the other hand, alludes to psychological, cultural and social differences between men and women [2].

Attitudes towards sexuality have always been related to the social era we are living in [3]. Likewise, sexual behaviors have been valued differently depending on whether they are related to men or women, even finding differences in child-rearing according to sex [4]. In relation to sexual behavior, men tend to have sex earlier, have more sexual partners, and have more casual partners, while women tend to initiate sex once they have started a long-term relationship associated with love [5,6,7].

Sexuality is seen by most people as something essential in their lives [8], then, it becomes necessary for achieving self-balance [9]. Thus, ensuring safe sexuality is becoming a core social objective, thereby identifying sexual violence as a public health issue [10].

Sexual violence involves actions ranging from verbal harassment to forced penetration, and a wide variety of types of coercion, from social pressure and intimidation to physical force [11]. The World Health Organization, WHO [12], certifies that sexual violence is any act that is sexually undesired by a person who receives it from another person in any environment; and, therefore, sexual violence is recognized as a public health problem [13]. The National Sexual Violence Resource Center (NSVRC) determines that sexual violence affects women, men and children throughout their lives. Thus, it is considered as a violation against the right to a safe life because a person is forced or manipulated to perform an unwanted sexual activity. The reasons for non-consent may include fear, age, illness, disability and/or the influence of drugs [14].

Sexual violence affects people of all genders, ages, religions, professions, ethnicities and sexual orientations [15]. However, social inequalities increase risk [16]. This is, in part, because of a belief in male-domination and romanticism, whose response is submission [17]. At some point in their lives, one out of six women have experienced an attempt of rape, 22% before the age of 12; and one out of 33 men, during their lives, have been victims of rape or attempted rape. Even, 75% before age 18 and 48% before age 12 [18].

Depending on how extreme the sexual violence is, it can be differentiated into three types: sexual harassment, sexual abuse or sexual assault. Sexual harassment involves the presence of verbal, non-verbal or written language requirements for sexual relations with a person who is rejecting them [19]; sexual abuse involves accessing another person’s body without consent and without physical violence (in minors, people with a disability, those who are drunk or drugged and therefore cannot authorize such sexual activity, etc.); and, sexual assault involves accessing another person’s body for an explicitly sexual activity, without consent and through violence [20].

Its most extreme form, though not the only one, is penetration [21]. While these are not the only types of sexual violence, there are other more sensitive ones that are not seen as rape, but they are intimidating and thus punishable, such as: incest, unwanted touching or sexual contact, showing one’s genitals or naked body to others without consent, sexual commercial exploitation, observing another person in a sexual or private act without their consent or knowledge, public masturbation, etc. [22]. The multiple and often highly sensitive ways in which sexual violence may take place means that victims themselves are often unaware that they have been sexually abused.

There are many reasons why people who are attacked in these circumstances do not report the facts [12], including shame, improper support systems, fear that the aggressor will take revenge, mistrust of authorities, pressure from others not to speak out because of “what they will say”, fear or risk of repercussions and being blamed [23].

Any type of violence may affect people’ daily lives, according to the National Sexual Violence Resource Center, which states that even if the event occurred years ago, they often experience changes in their emotional reactions (guilt, vulnerability, angry, confused, isolated), psychological reactions (nightmares, traumatic flashbacks, anxiety, low self-esteem) and physical reactions (physical damage, changes in eating and sleeping, or increased startle response).

Sexual violence can also generate other consequences which, according to Siria et al. [24], can include family and social problems, dropping out of school (in the case of minors), lost or neglected employment (in adults) andloss of willingness as part of the psychosocial problems related to this issue.

The aggressors tend to have high levels of masculinity [25]. This gender role is understood as a set of attributes, values, functions and behaviors, which are learned throughout life socially andempower the person with a dominant behavior in order to subordinate and underestimate the other gender and those belonging to the same for not embracing this behavior model [26]. Normally, it is men acting as aggressors towards women (due to the fact that men are seen as the strong sex), although there are several instances where women are the aggressors [27]. The victims of these aggressions tend to have low levels of masculinity and high levels of femininity, which, socially speaking, is known as a risk factor for vulnerability [28].

The impact of rape on sexual behavior tends to persist beyond the first year and it may produce multiple after-effects among the victims. It ranges from a decrease in sexual desire, interest and satisfaction to an increase in sexual dysfunction in intimate relationships, inducing fear and avoidance of these as all contact evokes memories of that aggression [29].

The general objective presented for this study is to analyze how the variables age, sex and having suffered abuse or violence may predict sexual satisfaction and sexual inhibition. In addition, we analyze the mediating role that the sexual role can play between these relationships. For this purpose, two mediational models will be developed based on the results obtained from four questionnaires, observing the differences between them.

The first model shows the mediational role of gender between sex, age and experience sexual abuse with the individual’s sexual satisfaction.

The second model shows how gender role modulates the relationship between the predictor variables described and sexual inhibition.

## 2. Materials and Methods

### 2.1. Participants

For this research, 403 students from the University of Jaén (Jaén, Spain) voluntarily participated. The ages of the students were between 18 and 45 years old (M: 22.26, SD: 3.20). Of the total of the participants 302 (74.9%) were women and 101 (25.1%) were men. These percentages are proportional to the distribution of gender in the total population of educators in Spain (National Statistics Institute, 2015). Participants belonged to the degrees of social education (39.21%), primary education (14.89%), psychology (19.35%), chemistry (10.17%), law (10.17%) and nursing (8.44%). This research has been approved by the Ethics Committee of the University of Jaén (DIC.18/8.PRY). We calculated the minimal sample size at 95% confidence level, with a 5% confidence interval at 80% of statistical power. In this regard, the estimated minimum sample size was 385.

### 2.2. Instruments

The research is made up of four scales:

- Bem Sex Role Inventory (BSRI-12). A reduced version of the popular BSRI [30] was used in this study to assess sexual role. BSRI-12 [31] measures the gender role using 12 items. Using these items, instrumental (men, items 2, 4, 6, 8, 10 and 12) and expressive traits (women, Items 1, 3, 5, 7, 9 and 11) are evaluated. Using a 7-point Likert scale, the participants had to indicate how they identified themselves according to each item, where 1 indicated “strongly disagree” and 7 indicated “strongly agree”.
- Sociosexual Orientation Inventory (SOI-R). The sociosexual orientation of the participants was evaluated with SOI-R [32]. This scale, using 9 items, measures 3 factors: behavior, attitude and sociosexual desire. The first three items evaluate lived sociosexual behavior (SOI-B), the next three evaluate attitudes towards casual sex (SOI-A) and the last three evaluate the frequency of sexual desires/fantasies (SOI-D). For the SOI-B items, a 9-point scale is used where sexual behavior is evaluated from 0 to 20, where the first option is “0 times” and 9 is “20 or more times”. For the rest of the items, a 7-point Likert scale is used where 1 means “Strongly disagree”, and 7 “Strongly agree”.
- Inhibited Sexual Desire Test (ISD). Lack of sexual desire is defined as the absence or decrease of sexual fantasies or desires and activity [33]. To evaluate inhibited sexual desire, we used the adaptation of the original scale [33] to Spanish made by Sierra et al. [34]. This scale has a total of 15 items using a 7-point Likert scale, with 1 being “totally false” and 7 “totally true”.
- New Sexual Satisfaction Scale (NESS). This scale aims to assess personal sexual satisfaction and that enjoyed by a partner (stable or spontaneous). This scale presents a new perspective of sexual satisfaction incorporating current contexts, such as social networks [35]. The scale consists of 20 items that assess satisfaction using a Likert-type scale, with 1 being “not at all satisfied” and 7 “totally satisfied”.

### 2.3. Data Analysis

All analysis in this study was conducted with R software. The α value for all statistical tests was set to 0.05. Data ccreening was performed before the factorial analysis to evaluate the distribution of data and assumptions. Before the treatment of the data obtained with the scales, the validity and internal consistency of the scales was verified by confirmatory factor analysis. Confirmatory factorial analysis (CFA) was conducted with lavaan R package [36]. The semTools package has been used to calculate composite reliability (CR) and average variance extracted (AVE). Diagonally weighted least squares (DWLS) was used as an estimation method for CFA to account for multivariate non-normality. Cronbach’s alpha and McDonald ω were used to assess reliability [37]. Once the factorial treatment was applied to the results of the scales, the scores given by the participants were scaled by the factor load resulting from the CFA, that is, the raw scores given by the participants to each item were multiplied by the standardized factor loads of each item [38]. To predict which factors could predict the scores expressed on the different scales, we performed a multiple regression analysis with the scaled scores obtained on each scale. Predictors were age, sex and having suffered physical y/or sexual abuse/violence. Finally, a mediational model is proposed to analyze the mediating role that the sexual role shows between sex and having suffered abuse/violenzce and inhibited sexual desire (ISD), on the one hand, and sexual satisfaction (NSSS) on the other. Mediational analysis was carried out using the medmad package from jamovi [39].

## 3. Results

The data screening carried out before the factorial treatment of the subscales showed that our data did not breach the assumption of additivity (no item showed multicollinearity r > 0.90, nor singularity r > 0.95). To analyze the assumptions of linearity, homogeneity and homoscedasticity, a regression was performed between our data and a set of random data. Later, we analyze the distribution of the residuals of the regression. The distribution of these residues was shown to be between −2 and +2, which does not meet the assumptions.

### 3.1. Analysis of the Subscales

To confirm the validity and internal structure of the scales used, a confirmatory factor analysis (CFA) was performed with the data obtained for each scale.

- Bem Sex Role Inventory (BSRI-12).

The CAF for BSRI-12 scale shows an excellent fit [40], χ_2_ (53) = 153.63, *p* < 0.001, con CFI = 0.934, TLI = 0.917, SRMR = 0.077, RMSEA = 0.069 (RMSEA 90% CI [0.056, 0.082]). The reliability of this scale was Cronbach’s α = 0.733 and McDonald’s ω = 0.754. We obtained a CR = 0.75 and AVE = 0.38 for BEM-M, y CR = 0.84 and AVE = 0.50 for BEM-F.

- Sociosexual Orientation Inventory (SOI).

The CAF for SOI scale shows an excellent fit [40], χ_2_ (24) = 27.097, *p* = 0.300, con CFI = 0.998, TLI = 0.997, SRMR = 0.046, RMSEA = 0.018 (RMSEA 90% CI [0.000, 0.046]). The reliability of this scale was Cronbach’s α = 0.848 and McDonald’s ω = 0.850. We obtained a CR = 0.80 and AVE = 0.60 for SOI-B, CR = 0.75 and AVE = 0.57 for SOI-A, and CR = 0.86 and AVE = 0.67 for SOI-D.

- Inhibited sexual desire test (ISD).

The CAF for ISD scale shows an excellent fit [40], χ_2_ (90) = 143.289, *p* < 0.001, con CFI = 0.960, TLI = 0.953, SRMR = 0.069, RMSEA = 0.038 (RMSEA 90% CI [0.026, 0.050]). The reliability of this scale was Cronbach’s α = 0.752 and McDonald’s ω = 0.793. For ISD we obtained a CR = 0.77 and AVE = 0.20.

- New Scale of Sexual Satisfaction (NSSS).

The CAF for ISD scale shows an excellent fit [40], χ_2_ (170) = 120.855, *p* = 0.998, con CFI = 1.00, TLI = 1.00, SRMR = 0.060, RMSEA = 0.000 (RMSEA 90% CI [0.000, 0.000]). The reliability of this scale was Cronbach’s α = 0.955 and McDonald’s ω = 0.956. The CR was 0.95 and AVE = 0.51 for NSSS.

### 3.2. Prediction Analysis (Multiple Regression)

Table 1 presents the results of the multiple regression analysis with the scaled scores obtained with each scale, for the predictors’ sex, abuse/violence and age.

As it can be seen, no variable predicts female behaviors (BEM-F). On the contrary, the results reveal that sex (β = −0.313), age (β = −0.116) and having suffered aggression/violence (β = 0.413) are good predictors of male behaviors (BEM-M). This means that men showed higher scores in BEM-M than women and as the age of the respondents increased, BEM-M scores also increased, and that people who indicated that they had suffered abuse/violence showed scores in BEM-M higher than those who answered no or maybe.

The results related to the scores obtained in SOI showed that sex (males higher scores than females, β = −0.235), and having suffered abuse/violence (higher scores for the condition Yes, β = 0.351) were predictors of SOI-B.

In the case of SOI-A, both sex (males higher scores than females, β = −0.555), as well as having suffered abuse/violence (higher scores for the condition Yes, β = 0.345), and age (scores decreased with increasing age, β = −0.111) were good predictors.

For SOI-D, sex (males scored higher than females, β = −0.874) and having suffered abuse/violence were good predictors (higher scores for the condition Yes, β = 0.360). For the ISD scores, none of the variables were good predictors. Finally, only sex showed to be a predictor for the scores obtained in NSSS (β = 0.302).

### 3.3. Mediational Analysis

**Figure 1:**
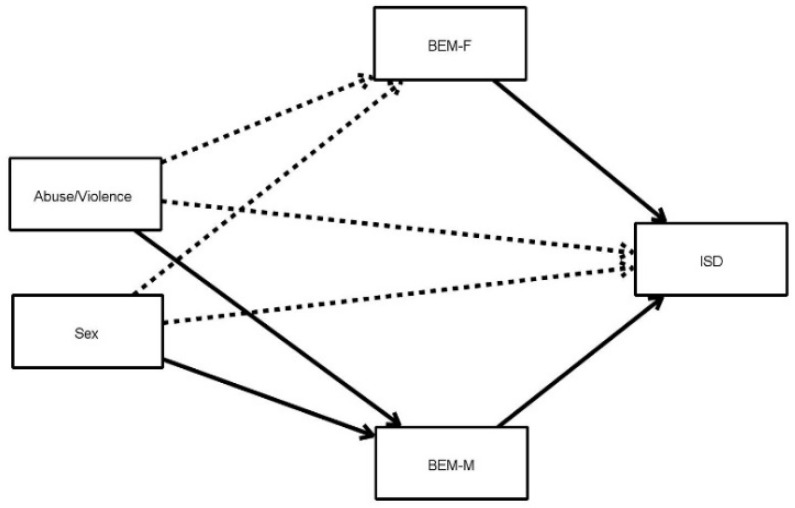
Mediational model. Categorical independent variables (factors) are shown with only one rectangle, but their effect is estimated using contrast variables. BEM-M = Male behaviors, BEM-F = Female behaviors; ISD = Inhibited sexual desire.

Figure 1 shows the proposed mediational model to understand how the sexual role can modulate the relationship between sex, and having suffered abuse/violence, with on the one hand inhibited sexual desire (upper part) and on the other hand sexual satisfaction (bottom).

The relationships that were significant in the analysis are represented in the figure by black arrows, while the non-significant relationships are represented by dotted arrows.

**Figure 2:**
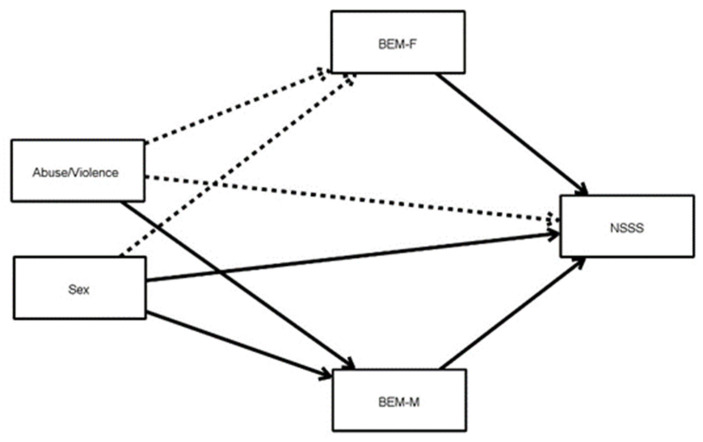
Mediational model. Categorical independent variables (factors) are shown with only one rectangle, but their effect is estimated using contrast variables. BEM-M = Male behaviors, BEM-F = Female behaviors; NSSS = New Scale of Sexual Satisfaction.

Table 2 shows all the results corresponding to the direct and indirect relationships in the two models proposed in Figure 1 and Figure 2.

As can be seen, male behavior (BEM-M) mediates the relationship between abuse/violence and inhibited sexual desire (β = −0.028), when the respondent claims to have suffered abuse/violence (condition abuse/violence1 in Table 2). This masculine behavior also shows mediating between sex (in the case of females, Sex1) and inhibited sexual desire (β = 0.028). Additionally, female behavior (BEM-F) showed a negative relationship with inhibited sexual desire (β = −0.175), see Table 2 for more details.

In the case of sexual satisfaction (represented in the lower part of Figure 1), male behavior (BEM-M) also mediates between abuse/violence and sexual satisfaction (NSSS) for the condition abuse/violence1 (when they answered Yes to the rest of conditions), β = 0.026. In this case, male behavior also showed mediating the relationship between sex and sexual satisfaction when sex was female (β = −0.027). Additionally, sex and female behavior showed a significant relationship with sexual satisfaction (β = −0.150 and β = 0.234, respectively); see Table 2 for more details.

## 4. Discussion

This research was intended to analyze the direct and mediating relationships for two models. In one of them, the dependent variable that we try to explain is sexual inhibition and in the other one is sexual satisfaction among university students. Likewise, and taking into account the characteristics of the university stage, it refers to a consistent lifetime where personality and gender roles tend to be maintained over time, as opposed to lower stages as suggested by Donelly and Twenge [41].

Similar to other studies, participants’ sex is not an indicator of gender role [42]. Among other reasons, this may be due to the need to adjust gender roles to the social norms, beliefs and attitudes prevalent in today’s society [2,43].

Androgyny, understood in terms of the concentration of male and female traits regardless of sex and stereotypes [2,44], is another factor to consider in the interpretation of the results obtained. Many studies have indicated that social changes and the readjustment of gender roles, especially those of women [45], have been influenced by factors such as the incorporation of women into the world of work or the assumption of child-rearing tasks by men that have broken the conception of the old male–female dichotomy are insufficient in gender classification [46]. 

These findings report that sex, age and experience as a victim of sexual assault are predictors of male role, unlike the Ferrer-Perez and Bosch-Fiol study [47], which found no significant association between sex and sexual profiling.

In terms of commitment, this research has shown that men tend to score higher on the SOI scale; this implies that they do not require commitment to the other person in order to have sex [48]. These results are consistent with the study by Petersen and Hyde [49], who suggest that men are more receptive than women to casual sex. Similarly, changing attitudes that include sex life, gender roles, and current patterns and lifestyles indicate an increasing trend of sexual assault or attempted sexual assault among youth. Specifically to the American college population, it is estimated that one out of five women and one out of 20 men will be victims of attempted or completed sexual assault [50].

Attitude toward sex, along with the frequency of sexual fantasies, are other aspects closely related to sociosexual behavior [51]; both factors were analyzed in the SOI scale [32]. In the present study, the data reveals how men tend to have more male features, in terms of having more sexual fantasies and having more active sexual behavior, in line with specialized research on the subject [52].

On the other hand, the continuous efforts to achieve equity in terms of gender roles have led to the emergence of manuals aimed at empowering women’s sexuality in terms of gender [52]. However, morality often limits women in terms of sexual freedom or casual sex. 

These issues also justify the results obtained in terms of sexual inhibition and gender role. Based on what has been obtained, it has been found that people who have experienced episodes of sexual violence tend to be more sexually inhibited than those who do not know or deny having experienced them. Depending on the sex, no differences were found between men and women in terms of sexual inhibition, as was the case in the study by Sierra et al. [34]. In the examination of gender roles, it has been found that people who display a greater number of male features tend to be less inhibited [53,54,55].

Despite the findings we achieved, it is important to point out that this research has some limitations that should be taken into account. Firstly, there is a limitation related to the sample. In our study, 403 university students from a single university participated. Similarly, the superiority of women over men in the sample means that the results should be interpreted with caution. In addition, a cross-sectional study means that the results presented here should be assumed under caution. Further studies will extend the sample to different contexts, with a view to determine whether this trend is generalized or not. Similarly, longitudinal research would contribute to a better explanation of the reality studied.

## 5. Conclusions

Nowadays, it is more pertinent to talk about gender rather than sex to establish behavioral patterns in order to establish a more inclusive language. Also, people who exhibit masculine features, regardless of sex, tend to feel more sexually satisfied. They also have a more open sexuality than those with female features.

It can be concluded that people who have been sexually harassed or assaulted have more masculine features than those who do not know or have not been abused. Likewise, age proves to be a good predictor of male features, since as it increases, the person’s masculine features become greater.

With respect to the sexual inhibition variable, victims of sexual abuse present higher inhibition rates than those who have not suffered abuse or do not know they have suffered it. Finally, and as a future line of research, it is necessary to delve into the causes that motivate the sexual inhibition of people who have greater female features than men.

## Figures and Tables

**Table 1:** Predictors for multiple regression.

							95% CI	
	Predictor	Estimate	SE	*t*	*p*	β	Lower	Upper
BEM-F	Intercept	3.671	0.221	16.622	<0.001			
	Sex:							
	Woman-Men	0.047	0.072	0.651	0.515	0.078	−0.158	0.314
	Abuse/Violence:							
	Yes–No	0.132	0.084	1.577	0.115	0.221	−0.054	0.496
	Maybe–No	0.134	0.095	1.410	0.159	0.224	−0.088	0.536
	Age	−0.001	0.009	−0.144	0.885	−0.007	−0.109	0.094
BEM-M	Intercept	2.138	0.201	10.650	<0.001			
	Sex:							
	Woman-Men	−0.174	0.065	−2.674	0.008	−0.313	−0.543	−0.083
	Abuse/Violence:							
	Yes–No	0.230	0.076	3.032	0.003	0.413	0.145	0.681
	Maybe–No	−0.029	0.086	−0.339	0.735	−0.052	−0.357	0.252
	Age	0.020	0.009	2.308	0.022	0.116	0.017	0.215
SOI-B	Intercept	1.211	0.396	3.056	0.002			
	Sex:							
	Woman-Men	−0.255	0.129	−1.979	0.049	−0.235	−0.469	−0.002
	Abuse/Violence:							
	Yes–No	0.381	0.150	2.538	0.012	0.351	0.079	0.624
	Maybe–No	0.062	0.170	0.364	0.716	0.057	−0.252	0.366
	Age	0.024	0.017	1.405	0.161	0.072	−0.029	0.172
SOI-A	Intercept	3.463	0.348	9.963	<0.001			
	Sex:							
	Woman-Men	−0.537	0.113	−4.761	<0.001	−0.555	−0.785	−0.326
	Abuse/Violence:							
	Yes–No	0.334	0.132	2.541	0.011	0.345	0.078	0.613
	Maybe–No	−0.049	0.149	−0.325	0.745	−0.050	−0.354	0.253
	Age	−0.033	0.015	−2.219	0.027	−0.111	−0.210	−0.013
SOI-D	Intercept	3.497	0.440	7.954	<0.001			
	Sex:							
	Woman-Men	−1.115	0.143	−7.812	<0.001	−0.874	−1.094	−0.654
	Abuse/Violence:							
	Yes–No	0.459	0.166	2.761	0.006	0.360	0.104	0.616
	Maybe–No	0.060	0.189	0.320	0.749	0.047	−0.244	0.338
	Age	−0.023	0.019	−1.195	0.233	−0.057	−0.152	0.037
ISD	Intercept	1.023	0.129	7.954	<0.001			
	Sex:							
	Woman-Men	0.011	0.042	0.252	0.801	0.030	−0.207	0.268
	Abuse/Violence:							
	Yes–No	−0.020	0.049	−0.421	0.674	−0.059	−0.336	0.217
	Maybe–No	0.007	0.055	0.119	0.906	0.019	−0.295	0.333
	Age	−0.004	0.006	−0.784	0.433	−0.041	−0.143	0.061
NSSS	Intercept	4.448	0.269	16.539	<0.001			
	Sex:							
	Woman-Men	0.222	0.087	2.542	0.011	0.302	0.069	0.536
	Abuse/Violence:							
	Yes–No	0.130	0.102	1.278	0.202	0.177	−0.095	0.449
	Maybe–No	−0.049	0.116	−0.423	0.672	−0.067	−0.376	0.243
	Age	−0.018	0.012	−1.537	0.125	−0.079	−0.179	0.022

Notes. BEM-M = Male behaviors, BEM-F = Female behaviors; SOI-B = Sociosexual Orientation Inventory-Behavior, SOI-A = Sociosexual Orientation Inventory-Attitude, SOI-D = Sociosexual Orientation Inventory-Desire; ISD = Inhibited sexual desire; NSSS = New Scale of Sexual Satisfaction.

**Table 2:** Indirect and total effect from mediation analysis for ISD and NSSS.

					95% C.I. (a)				
	Type	Effect	Estimate	SE	Lower	Upper	β	*z*	*p*
ISD	Indirect	Abuse/violence1⇒BEM-F⇒ISD	−0.013	0.009	−0.031	0.004	−0.014	−14.487	0.147
		Abuse/violence1⇒BEM-M⇒ISD	−0.026	0.011	−0.048	−0.004	−0.028	−23.933	0.017
		Abuse/violence2⇒BEM-F⇒ISD	−0.013	0.010	−0.033	0.006	−0.012	−13.257	0.185
		Abuse/violence2⇒BEM-M⇒ISD	0.003	0.009	−0.014	0.022	0.003	0.4188	0.675
		Sex1⇒BEM-F⇒ISD	−0.005	0.007	−0.019	0.009	−0.006	−0.6988	0.485
		Sex1⇒BEM-M⇒ISD	0.022	0.009	0.004	0.041	0.028	24.257	0.015
	Component	Abuse/violence1⇒BEM-F	0.130	0.082	−0.031	0.292	0.079	15.806	0.114
		BEM-F⇒ISD	−0.101	0.028	−0.157	−0.046	−0.175	−36.220	<0.001
		Abuse/violence1⇒BEM-M	0.246	0.075	0.097	0.394	0.160	32.528	0.001
		BEM-M⇒ISD	−0.108	0.030	−0.168	−0.048	−0.175	−35.340	<0.001
		Abuse/violence2⇒BEM-F	0.134	0.094	−0.050	0.318	0.072	14.246	0.154
		Abuse/violence2⇒ BEM-M	−0.036	0.086	−0.205	0.132	−0.020	−0.421	0.673
		Sex1⇒BEM-F	0.049	0.069	−0.086	0.184	0.035	0.712	0.476
		Sex1⇒BEM-M	−0.210	0.063	−0.334	−0.087	−0.164	−33.35	<0.001
	Direct	Abuse/violence1⇒ISD	0.016	0.047	−0.076	0.109	0.016	0.339	0.734
		Abuse/violence2⇒ISD	0.017	0.053	−0.086	0.122	0.016	0.334	0.738
		Sex1⇒ISD	0.000	0.039	−0.077	0.078	0.000	0.014	0.988
	Total	Abuse/violence1⇒ISD	−0.023	0.048	−0.118	0.070	−0.025	−0.495	0.620
		Abuse/violence2⇒ISD	0.008	0.055	−0.099	0.115	0.007	0.147	0.883
		Sex1⇒ISD	0.018	0.040	−0.060	0.097	0.023	0.458	0.647
NSSS	Indirect	Abuse/violence1⇒BEM-F⇒NSSS	0.037	0.025	−0.011	0.086	0.018	1.506	0.132
		Abuse/violence1⇒BEM-M⇒NSSS	0.054	0.022	0.009	0.098	0.026	2.370	0.018
		Abuse/violence2⇒BEM-F⇒NSSS	0.038	0.028	−0.016	0.094	0.016	1.369	0.171
		Abuse/violence2⇒BEM-M⇒NSSS	−0.007	0.019	−0.045	0.029	−0.003	−0.419	0.675
		Sex1⇒BEM-F⇒NSSS	0.014	0.020	−0.025	0.053	0.008	0.705	0.481
		Sex1⇒BEM-M⇒NSSS	−0.046	0.019	−0.084	−0.008	−0.027	−2.401	0.016
	Component	Abuse/violence1⇒BEM-F	0.130	0.082	−0.031	0.292	0.079	1.581	0.114
		BEM-F⇒NSSS	0.288	0.058	0.174	0.402	0.234	4.963	<0.001
		Abuse/violence1⇒BEM-M	0.246	0.075	0.097	0.394	0.160	3.253	0.001
		BEM-M⇒NSSS	0.219	0.063	0.095	0.344	0.167	3.459	<0.001
		Abuse/violence2⇒BEM-F	0.134	0.094	−0.050	0.318	0.072	1.425	0.154
		Abuse/violence2⇒ BEM-M	−0.036	0.086	−0.205	0.132	−0.020	−0.422	0.673
		Sex1⇒BEM-F	0.049	0.069	−0.086	0.184	0.035	0.712	0.476
		Sex1⇒BEM-M	−0.210	0.063	−0.334	−0.087	−0.164	−3.336	<0.001
	Direct	Abuse/violence1⇒NSSS	0.024	0.098	−0.168	0.216	0.011	0.245	0.806
		Abuse/violence2⇒NSSS	−0.073	0.110	−0.289	0.142	−0.032	−0.664	0.507
		Sex1⇒NSSS	0.286	0.081	0.126	0.447	0.169	3.504	<0.001
	Total	Abuse/violence1⇒NSSS	0.115	0.101	−0.082	0.314	0.057	1.146	0.252
		Abuse/violence2⇒NSSS	−0.042	0.115	−0.268	0.183	−0.018	−0.369	0.712
		Sex1⇒NSSS	0.254	0.084	0.089	0.420	0.150	3.013	0.003

Notes. BEM-M = Male behaviors, BEM-F = Female behaviors; ISD = Inhibited sexual desire; NSSS = New Scale of Sexual Satisfaction. For variable Abuse/violence the contrasts are: Abuse/violence1—Yes—No, Abuse/violence2—Maybe—No. For variable Sex, the contrasts are: Sexo1—Women—Men. (a) Confidence intervals computed with method: Standard Delta method.

## Data Availability

The data are available for anyone who wants to see them with justified reasons. Please contact the correspondence author.
